# Prediction of sensitivity to gefitinib/erlotinib for EGFR mutations in NSCLC based on structural interaction fingerprints and multilinear principal component analysis

**DOI:** 10.1186/s12859-018-2093-6

**Published:** 2018-03-07

**Authors:** Bin Zou, Victor H. F. Lee, Hong Yan

**Affiliations:** 1 Department of Electronic Engineering, City University of Hong Kong, Kowloon, Hong Kong, China; 2 Department of Clinical Oncology, Li Ka Shing Faculty of Medicine, The University of Hong Kong, Pokfulam, Hong Kong, China

**Keywords:** Epidermal growth factor receptor mutation, Molecular dynamics simulations, Interaction fingerprints, Multilinear principal component analysis

## Abstract

**Background:**

Non-small cell lung cancer (NSCLC) with activating EGFR mutations, especially exon 19 deletions and the L858R point mutation, is particularly responsive to gefitinib and erlotinib. However, the sensitivity varies for less common and rare EGFR mutations. There are various explanations for the low sensitivity of EGFR exon 20 insertions and the exon 20 T790 M point mutation to gefitinib/erlotinib. However, few studies discuss, from a structural perspective, why less common mutations, like G719X and L861Q, have moderate sensitivity to gefitinib/erlotinib.

**Results:**

To decode the drug sensitivity/selectivity of EGFR mutants, it is important to analyze the interaction between EGFR mutants and EGFR inhibitors. In this paper, the 30 most common EGFR mutants were selected and the technique of protein-ligand interaction fingerprint (IFP) was applied to analyze and compare the binding modes of EGFR mutant-gefitinib/erlotinib complexes. Molecular dynamics simulations were employed to obtain the dynamic trajectory and a matrix of IFPs for each EGFR mutant-inhibitor complex. Multilinear Principal Component Analysis (MPCA) was applied for dimensionality reduction and feature selection. The selected features were further analyzed for use as a drug sensitivity predictor. The results showed that the accuracy of prediction of drug sensitivity was very high for both gefitinib and erlotinib. Targeted Projection Pursuit (TPP) was used to show that the data points can be easily separated based on their sensitivities to gefetinib/erlotinib.

**Conclusions:**

We can conclude that the IFP features of EGFR mutant-TKI complexes and the MPCA-based tensor object feature extraction are useful to predict the drug sensitivity of EGFR mutants. The findings provide new insights for studying and predicting drug resistance/sensitivity of EGFR mutations in NSCLC and can be beneficial to the design of future targeted therapies and innovative drug discovery.

**Electronic supplementary material:**

The online version of this article (10.1186/s12859-018-2093-6) contains supplementary material, which is available to authorized users.

## Background

Somatic mutations in the kinase domain of the epidermal growth factor receptor (EGFR) gene are detected in about 10–35% of patients with advanced non-small cell lung cancer (NSCLC) [1–3]. These mutations occur within EGFR exons 18–21 and more than 80% of them are exon 19 deletions or the exon 21 L858R point mutation [4, 5]. The first-generation EGFR tyrosine kinase inhibitors (TKI), including gefitinib and erlotinib, which reversibly bind to the kinase domain of EGFR, are widely used to treat NSCLC patients with activating EGFR mutations [6–13]. These inhibitors block the abnormal subsequent signal transduction caused by EGFR mutations and lead to inhibition of tumor proliferation.

Tumors with activating EGFR mutations, especially exon 19 deletions and the L858R point mutation, are particularly responsive to gefitinib and erlotinib, with an objective response rate (ORR) of approximately 60% [7, 8, 11–13]. However, the sensitivity varies for less common and rare EGFR mutations. Most EGFR exon 20 insertions except A763_Y764insFQEA (about 4.0–9.2% of all lung tumors with EGFR mutations [4, 14–17]), the exon 20 T790 M point mutation (in less than 5% of untreated tumors [18] and over 50% of treated tumors that have acquired resistance to gefitinib/erlotinib [19, 20]), and the complex mutations L858R/T790 M and exon 19 deletion/T790 M, are associated with low sensitivity to clinically achievable doses of gefitinib/erlotinib. Some other less common mutations, like exon 18 point mutations in position G719 (G719A, C or S, about 3% of all tumors) and the exon 21 L861Q mutation (about 2% of all tumors), are associated with some level of sensitivity to gefitinib/erlotinib [1, 4, 21–30].

There are various explanations for the different sensitivities of EGFR mutations to gefitinib/erlotinib. For the T790 M mutation, two possibilities were raised. One is that substitution of threonine 790 with a bulky methionine sterically interferes with the binding of TKIs [19, 20, 31]. Another is that introduction of the T790 M mutation increases the affinity for adenosine triphosphate (ATP) which reduces binding of competing TKIs such as gefitinib and erlotinib [19, 20, 32]. For EGFR exon 20 insertions, one explanation is that the insertion forms a “wedge” at the end of the C-helix that may effectively lock the helix in its active position [17]. However, there are few structural studies on less common mutations, such as G719X and L861Q that still demonstrate some sensitivity to gefitinib/erlotinib. Our group has previously attempted to decipher the mechanism of drug resistance based on several computational methods, including analysis of local surface geometric properties [33–35], binding free energy [34, 36] and stability analysis [37]. These studies provided useful references to understand the sensitivity of EGFR mutants to gefitinib or erlotinib.

To decode the drug sensitivity or selectivity of EGFR mutants, it is important to analyze the interaction between EGFR mutants and EGFR inhibitors. Protein-ligand interaction fingerprint (IFP) based methods [38–40], which encode the protein-ligand interfacial interaction as 1D fingerprints, has been widely applied to protein-ligand interaction mining [41], binding site comparisons [39], prediction of binding mode [42] and other studies [43–46]. Thus, IFP should be a promising method to compare the binding mode of EGFR mutants with EGFR inhibitors. As proteins are always dynamic, with their atoms constantly in motion, the protein-ligand IFP will change overtime even if a protein is in a stable state. Therefore, each EGFR mutant-inhibitor complex will have multiple versions of its protein-ligand IFP. It is more reasonable to use these multiple versions of the IFP to depict the binding mode of one EGFR mutant-inhibitor complex.

In this study, we used the technique of IFP to analyze and compare the binding modes of EGFR mutants and EGFR inhibitors. Molecular dynamics simulations [47] were employed to obtain the dynamic trajectory and a matrix of IFP for each EGFR mutant-inhibitor complex. A Multilinear Principal Component Analysis (MPCA) framework [48] was applied for dimensionality reduction and feature selection. The selected features were further analyzed for use as a drug sensitivity predictor. Our results showed that the accuracy of prediction of drug sensitivity was very high for both gefitinib and erlotinib. The findings provide new insights into methods to study and predict drug resistance/sensitivity in lung cancer treatment and can guide future designs of targeted therapies and innovative drug discovery.

## Results

### EGFR mutation selection

EGFR mutations were selected according to the survey carried out in [49] and were the 11 most common exon 19 deletions, the 6 most common exon 20 insertions, the most common exon 18 deletion delE709_T710insD, the most common exon 19 insertion I744_K745insKIPVAI, G719X (A, C or S), E709X (A or K), S761I, L858R, L861Q and T790 M (including T790 M_L858R and T790 M_delE746_A750 complex mutations) (Table 1). These 30 mutations account for over 90% of all EGFR mutations.

**Table 1:** Selected EGFR mutations and their corresponding drug sensitivity to gefitinib/erlotinib based on the survey carried out by [49]

	Category	Mutations	Sensitivity
1	Del 19	delE746_A750	High
2		delL747_P753insS	
3		delL747_T751	
4		delL747_A750insP	
5		delL747_S752	
6		delE746_S752insV	
7		delE746_P753insVS	
8		delL747_T751insP	
9		delE746_T751insA	
10		delL747_P753	
11		delS752_I759	
12	L858R	L858R	
13	E709X	E709A	Moderate
14		E709K	
15	Del 18	delE709_T710insD	
16	G719X	G719A	
17		G719C	
18		G719S	
19	Ins 19	I744_K745insKIPVAI	
20	S768I	S768I	
21	L861Q	L861Q	
22	Ins 20	A763_Y764insFQEA	
23		V769_D770insASV	Low
24		D770_N771insSVD	
25		H773_V774insH	
26		H773_V774insPH	
27		H773_V774insNPH	
28	T790 M	T790 M	
29		T790 M_L858R	
30		T790 M_delE746_A750	

The sensitivities of the 30 EGFR mutations to gefitinib/erlotinib were divided into three levels, high, moderate, and low. This classification was done based on the data collected by [49] on in vitro sensitivities to gefitinib/erlotinib in Ba/F3 cells expressing each EGFR mutation. Specifically, exon 19 deletions and L858R have IC50 values (nM) of < 100. E709X (A or K), G719X (A, C or S), delE709_T710insD, I744_K745insKIPVAI, A763_Y764insFQEA, S768I and L861Q have IC50 values (nM) of 100–999. Other exon 20 insertions and T790 M (including T790 M_L858R and T790 M_delE746_A750) have IC50 values (nM) of > 1000. Sensitivity to gefitinib/erlotinib was then set as high, moderate and low, respectively.

### Computational simulation results

Although some EGFR mutant structures are available in the Protein Data Bank (PDB) [50], for example L858R-gefitinib (2ITZ) and G719S-gefitinib (2ITO), no structural information for most EGFR mutant-gefitinib/erlotinib complexes exists in the public domain. Most EGFR structural information in the PDB database is not completely recorded as some residues may not be seen in the electron density of the crystal structure. For example, residues 866–875 and 991–1001 of 2ITZ are not recorded. Therefore, computational modeling of the structures of all EGFR mutant-gefitinib/erlotinib complexes from a single template will be an appropriate approach. 1M17 (WT EGFR-erlotinib complex) was chosen as the template and the main part of the kinase domain (residues 696 to 988) was used.

**Fig. 1:**
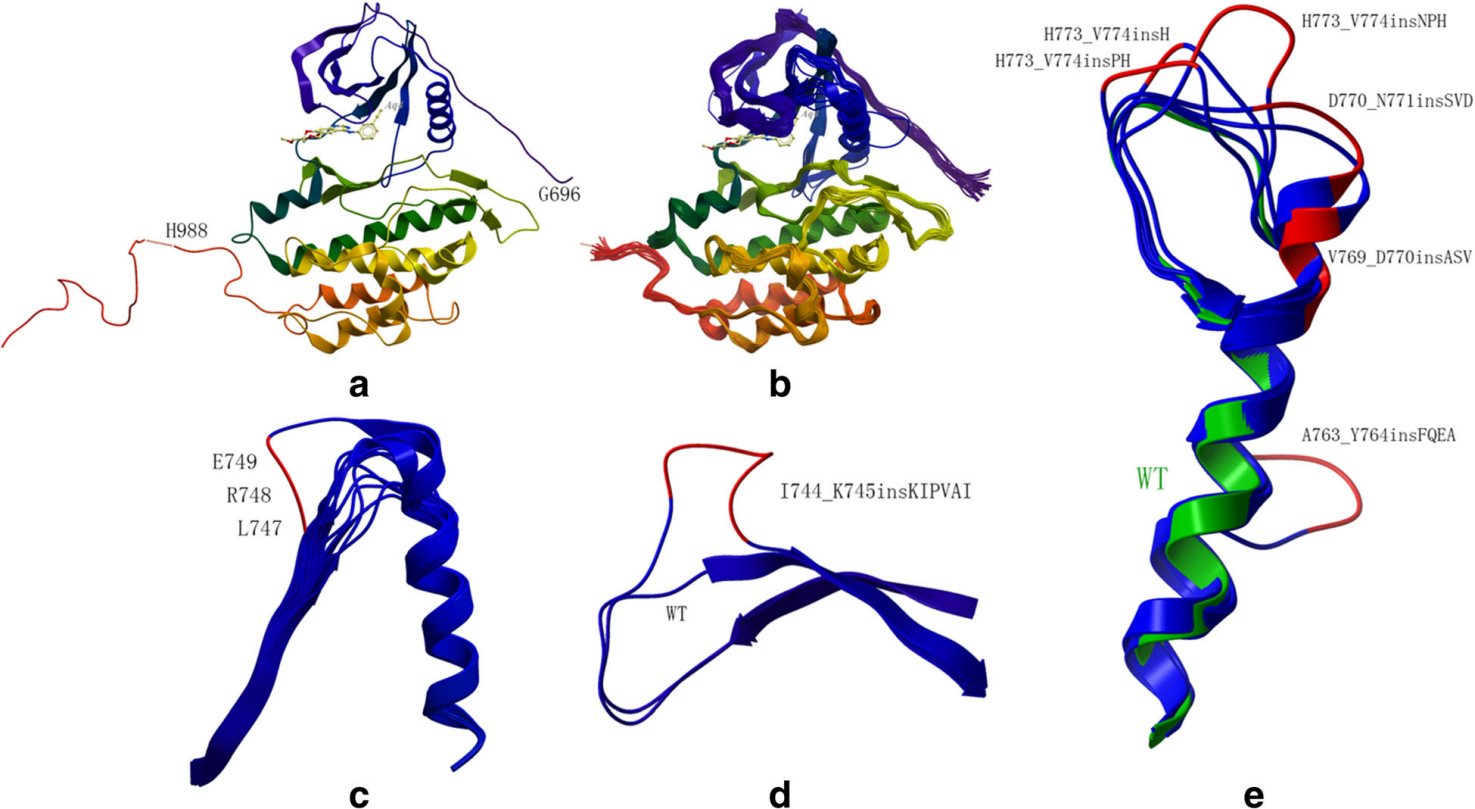
Computational modeling results of EGFR mutants. **a** The template WT EGFR structure (1M17). **b** All EGFR mutants involved. **c** Exon 19 deletions and WT EGFR structure. The three LRE residues are marked as red. **d** Exon 19 insertion I744_K745insKIPVAI and WT EGFR structure. The insertion site is marked as red. **e** Exon 20 insertions and WT EGFR structure. The insertion sites are marked as red and WT is marked as green

Structures for all EGFR mutants were generated using Rosetta and procedures similar to those described in [51] (Fig. 1). The structures of the EGFR mutants are very similar to that of WT EGFR (Fig. 1(b)) with differences in some mutants, especially exon 19 insertion I744_K745insKIPVAI, exon 19 deletions and exon 20 insertions (Fig. 1(c-e)). Compared with WT EGFR, the deletion and insertion sites of the mutants were rearranged. Only a small difference was observed in substitution mutants, like E709A, G719C and L858R.

Before performing MD simulations, EGFR mutants should be bound with gefitinib or erlotinib to generate EGFR mutant-gefitinib/erlotinib complexes. This was done based on structural alignment of the EGFR mutants to templates of EGFR-gefitinib (2ITY) or EGFR-erlotinib (1M17) complexes thus allowing proper placement of the TKI positions. After validating the equilibration of the system by observing the stability of the temperature, density, energy, and root mean square deviation (RMSD) of the system (see Additional file 1: Figure S1), MD simulations were performed and a trajectory of 1000 frames (2 ns) was obtained for each EGFR mutant-gefitinib/erlotinib complex.

### Interaction fingerprint calculation

**Fig. 2:**
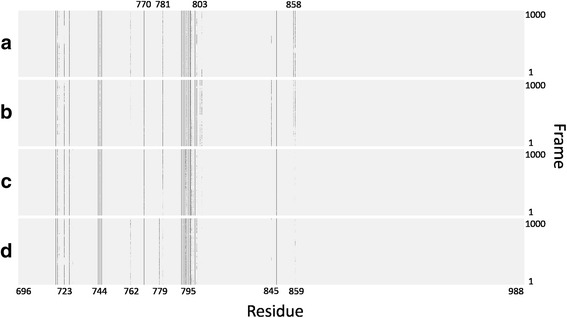
IFP matrices for four EGFR mutant-gefitinib complexes. **a** delE746_A750-gefitinib. **b** T790 M_delE746_A750-gefitinib. **c** A763_Y764insFQEA-gefitinib. **d** D770_N771insSVD-gefitinib

For each frame in the trajectory, we extracted its IFP and for all frames in the trajectory of each complex we produced an IFP matrix. This IFP matrix can be considered as the binding mode of this EGFR mutant with the specific TKI. Figure 2 shows the IFP matrices for four example EGFR mutant-gefitinib complexes, delE746_A750-gefitinib, T790 M_delE746_A750-gefitinib, A763_Y764insFQEA-gefitinib and D770_N771insSVD-gefitinib. Of these, delE746_A750 has high sensitivity to gefitinib, A763_Y764insFQEA has moderate sensitivity to gefitinib, while T790 M_delE746_A750 and D770_N771insSVD have low sensitivity to gefitinib.

In Fig. 2, the x-axis is the residue index and the y-axis is the frame number. Residues 723, 762, 779, 781, 803, 845, 858 and/or their neighboring residues have obvious differences among these four IFP matrixes. Even though differences between IFP matrixes can be seen, it is hard to conclude what kind of IFP matrix, or binding mode of an EGFR mutant-TKI complex, corresponds to high, moderate, or low sensitivity to gefitinib/erlotinib. One solution is to reduce the data dimensionality and extract the most discriminative features, which can be done by Multilinear Principal Component Analysis (MPCA).

### MPCA-based tensor objects recognition

With MPCA, a multilinear equivalent of PCA, we can determine a multilinear transformation that maps tensor objects onto a lower dimensional tensor subspace while preserving the variation in the original data. In this work, we applied the MPCA framework to extract features from the IFP matrix (2nd-order tensor) objects.

**Fig. 3:**
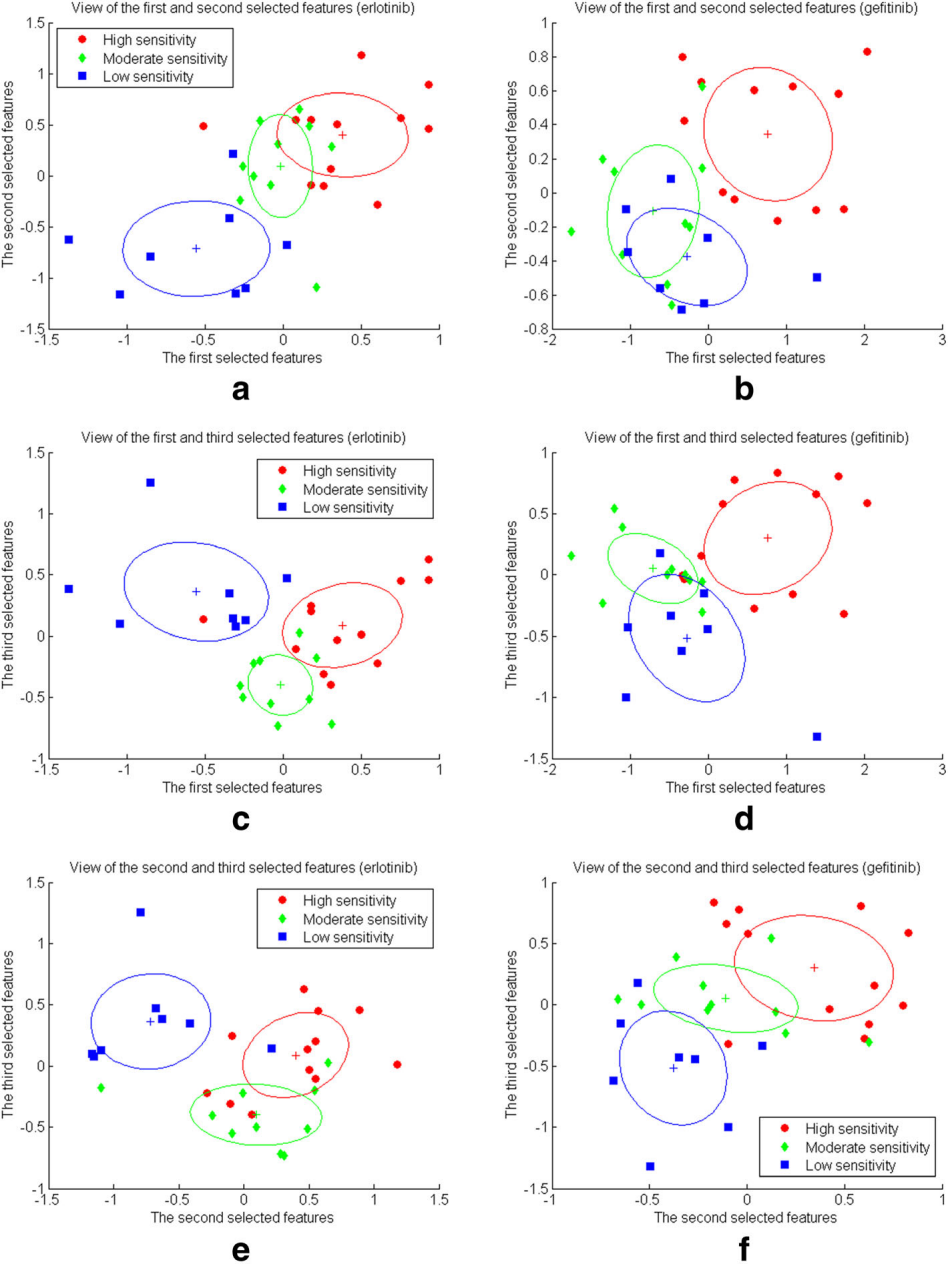
Distributions of EGFR mutant samples described with the first 3 selected features. **a**, **c** and **e** are for EGFR mutant-erlotinib complexes and **b**, **d** and **f** are for EGFR mutant-gefitinib complexes. **a** and **b** are projections of the mutant features to the first and second selected features. **c** and (d) are projections of the mutant features to the first and third selected features. **e** and **f** are projections of the mutant features to the second and third selected features. Here, red, green and blue circles represent mutant groups that correspond to high, moderate and low sensitivity to gefitinib / erlotinib, and’+’ stands for the centroid of each group

After combining the IFP matrixes of multiple EGFR mutant-TKI complexes, we can obtain a third order IFP tensor. Using this 3rd-order IFP tensor and the label of each EGFR mutant-TKI complex (the sensitivity to gefitinib/erlotinib) as inputs to the MPCA framework, we can produce a lower dimensional tensor, which is then rearranged into a feature vector, in descending order according to class discriminability, and the first H most discriminative components are kept and used as the extracted features. The value of H is empirically determined. In our work, as we had only 30 samples for each TKI, we used values of H from 3 to 20 for the drug sensitivity prediction task. Figure 3 shows the views of the first-second, first-third and second-third selected features for all EGFR mutant-gefitinib and –erlotinib complexes. We can see that the three mutant groups can be roughly separated using only the first three extracted features. The class discrimination power of projected tensor features is shown in Additional file 1: Figure S2 and the selected 20 features for EGFR mutant-gefitinib and -erlotinib complexes are shown in Additional file 2.

To verify that our extracted features are useful to predict the sensitivity to gefitinib/erlotinib of each EGFR mutant, we performed classification experiments using the 5 most commonly used classifiers available in Weka 3.8.0, NaiveBayes, Logistic (logistic regression), RandomForest, libSVM (Support Vector Machine) and IBK (KNN, k-Nearest Neighbor). For RandomForest, we set the number of iterations to be performed at 500. For IBK we set the number of neighbor to use at 5. All other parameters were left as default values.

**Fig. 4:**
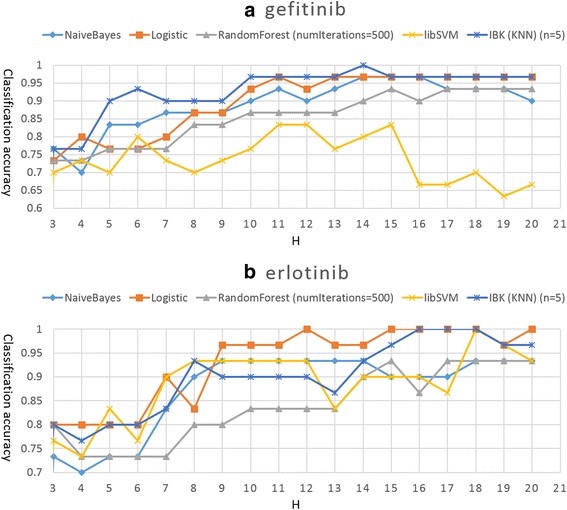
Classification accuracies of the five most commonly used classifiers against different values of H. H means the number of most discriminative components of the output feature vector retained for classification. **a** The classification accuracies for EGFR mutant-gefitinib complexes. **b** The classification accuracies for EGFR mutant-erlotinib complexes

The results are shown in Fig. 4. The x-axis is the value of H, which means the first H most discriminative components of the feature vector. The y-axis is the classification accuracy or the recognition rate. For the two groups of data (EGFR mutant-gefitinib and erlotinib complexes), the classification accuracies increase as H increases for most classifiers. When H equals 3, accuracies are about 75%, while at H equal to 9 or 10, accuracies reach about 90%. After that, accuracies remain at a high level except for libSVM with EGFR mutant-gefitinib complexes.

**Fig. 5:**
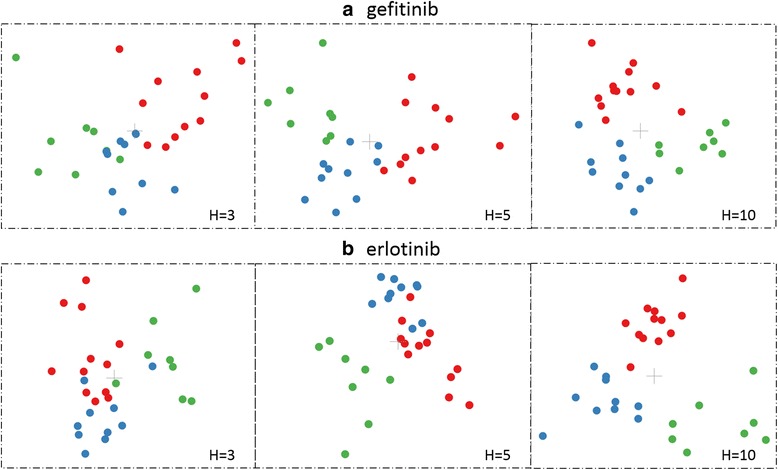
Data points with different values of H using targeted projection pursuit. **a** EGFR mutant-gefitinib complexes. **b** EGFR mutant-erlotinib complexes. Each point stands for an EGFR mutant-TKI complex with high (red) moderate (blue) or low (green) sensitivity to the corresponding TKI

We also used Targeted Projection Pursuit (TPP), an interactive data exploration technique that provides an intuitive and transparent interface for data exploration [52], to further verify the classification results. Views with three values of H, 3, 5 and 10, are presented for the two groups of data in Fig. 5. The three kinds of points (different drug sensitivities) separate more clearly as H increases. At H equal to 10, the three classes can be separated easily.

## Discussion

Tumors with activating EGFR mutations, especially exon 19 deletions and the L858R point mutation, are particularly responsive to gefitinib and erlotinib. However, the sensitivity varies for less common and rare EGFR mutations. There are various explanations for the low sensitivity of EGFR exon 20 insertions and the exon 20 T790 M point mutation to gefitinib/erlotinib. However, few studies discuss, from a structural perspective, why some less common mutations, like G719X and L861Q, have moderate sensitivity to gefitinib/erlotinib. To decode the drug sensitivity/selectivity of EGFR mutants, it is important to analyze the interaction between EGFR mutants and EGFR inhibitors.

In this study, we used IFP to analyze and compare the binding mode of EGFR mutant-inhibitor complexes, applied the MPCA framework to extract features from the IFP data and employed several commonly used classifiers to predict the sensitivity to gefitinib/erlotinib for each EGFR mutant. The 30 most common EGFR mutants were defined to have high, moderate or low sensitivity to gefitinib/erlotinib based on data collected by [49]. Structures for all EGFR mutant-inhibitor complexes were generated and MD simulations were used to produce a trajectory of 1000 frames (2 ns) for each EGFR mutant-gefitinib/erlotinib complex. The IFP for each frame in the trajectory was extracted to form an IFP matrix for the trajectory. This IFP matrix can be considered as the binding mode of this EGFR mutant with the specific TKI. MPCA was applied to extract features from the IFP matrix (2nd-order tensor) giving a feature vector for each EGFR mutant-inhibitor complex. To verify that the extracted features were useful to predict sensitivity to gefitinib/erlotinib for each EGFR mutant, classifications using the 5 most commonly used classifiers in Weka 3.8.0 were performed. The accuracy of the prediction of drug sensitivity was very high (> 90%) for both gefitinib and erlotinib. To verify the classification results and view the data points more clearly, Targeted Projection Pursuit (TPP) was used to show that the data points can be easily separated based on their sensitivities to gefitinib/erlotinib. Thus, the IFP features of EGFR mutant-TKI complexes and MPCA-based tensor object feature extraction are helpful to predict the drug sensitivity of EGFR mutants.

Our study has some limitations. First, only the 30 most common EGFR mutations of at least 594 types of EGFR mutations reported in the COSMIC database [53] were used. However, these 30 mutations account for more than 90% of all EGFR mutations. Sensitivity of the other mutations to gefitinib/erlotinib are not certain due to limited clinical data. The 30 most common mutations provide more reliable data for this study. Secondly, we determined sensitivity to gefitinib/erlotinib based on information from [49]. Specifically, for EGFR mutants with IC50 values (nM) of < 100, 100–999 and > 1000, sensitivity to gefitinib/erlotinib was set as high, moderate, or low, respectively. These IC50 values will have a certain amount of error. In one case, the IC50 values for delE746_S752insV showed a large difference – 306 with gefitinib and 14 with erlotinib. Sensitivity to gefitinib/erlotinib for this mutant was set to high as EGFR exon 19 deletions respond well to gefitinib/erlotinib. IC50 values are continuous and the choice of cut-off values (100 and 1000) may affect the classification accuracy. We believe that the influence will be small and our results are reliable as a whole. On the other hand, although gefitinib and erlotinib have different structures, different pharmacokinetic and pharmacodynamics properties and different affinities with their receptors, several studies [54–56] showed that they demonstrated comparable effects on progression-free survival, overall survival, overall response rate and disease control rate, which did not vary considerably with EGFR mutation status. Thus, we treated the sensitivity to gefitinib and erlotinib for each EGFR mutant as the same. The third limitation is that the method used in this study may be not suitable for irreversible TKIs, such as afatinib and osimertinib, because it is difficult to simulate the process of the formation of the covalent bond. It is not meaningful to study the binding mode of EGFR mutant and irreversible TKIs after the covalent bond has been formed. Other methods are needed to study irreversible TKIs.

Selection of the EGFR template structure to model the EGFR mutants may affect the results. A crystal structure of an active WT EGFR tyrosine kinase domain with gefitinib or erlotinib, of which there are - 1M17 (WT EGFR with erlotinib), 2ITY (WT EGFR with gefitinib) and 4WKQ (WT EGFR with gefitinib), is a reasonable template. 1M17 is the most complete structure with only residues 989 to 1000 missing in the electron density. Since residues after 988 are the ‘tail’ of the kinase domain and are far from the binding site, ignoring these residues is reasonable when modeling other EGFR mutants.

Although the MPCA framework and the five most common classifiers available in Weka 3.8.0 were chosen to study the performance of our proposed drug sensitivity prediction scheme, other feature extraction methods and classifiers could also be investigated to potentially improve the classification results.

## Conclusions

IFP was used to analyze and compare the binding mode of the 30 most common EGFR mutants with gefitinib or erlotinib. MPCA was used to extract features from the IFP data and several commonly used classifiers were employed to predict the sensitivity to gefitinib/erlotinib for each EGFR mutant. A high accuracy in prediction of sensitivity to gefitinib and erlotinib was obtained. By visualizing the data points using Targeted Projection Pursuit (TPP), the data points could be easily separated according to their sensitivities to gefitinib/erlotinib. Thus, we can conclude that the IFP features of EGFR mutant-TKI complexes and the MPCA-based tensor object feature extraction are helpful to predict the drug sensitivity of the relatively rarer EGFR mutants. The findings here can provide new insights for studying and predicting drug resistance/sensitivity of EGFR mutations in NSCLC treatment and can be beneficial to the design of future targeted therapies and innovative drug discovery.

## Methods

### Computer simulation

#### A. EGFR mutant-TKI complex modeling

Our method for EGFR mutant-TKI complex modeling consisted of three main steps. The first step was to choose a template structure of the WT EGFR kinase domain. In this study, 1M17 (EGFR WT-erlotinib complex) was chosen and the main part of the kinase domain (residues 696 to 988) was used as the template.

The second step was to generate structures for all EGFR mutants using Rosetta [57] and the procedures were similar to those described in [51]. Specifically, EGFR point mutants were generated using the Rosetta ddg_monomer protocol. EGFR deletions and insertions were generated using the Rosetta comparative modeling (CM) protocol. We also performed an energy minimization using Amber to optimize the generated structures [58].

The third step was to combine the above EGFR mutant structures with gefitinib or erlotinib to generate EGFR mutant-gefitinib/erlotinib complexes. This was done through structural alignment using Molsoft ICM-Browser (http://www.molsoft.com/icm_browser.html) [59]. Specifically, the EGFR mutant structures were aligned to templates of the EGFR-gefitinib (2ITY) or EGFR-erlotinib (1M17) complexes. Then the positions of the gefitinib of 2ITY or the erlotinib of 1M17 were taken to obtain EGFR mutant-gefitinib/erlotinib complexes. An energy minimization was performed on the structures to remove possible conflicts between the EGFR mutants and gefitinib/erlotinib.

#### B. Molecular dynamics (MD) simulations

Amber 16 was used to perform MD simulations [58]. Before performing the key production MD simulations, two more steps were needed - preparation of the coordinate (.inpcrd) and topology (.prmtop) files of the EGFR mutant-TKI complexes and minimization and equilibration of the system to guarantee a stable simulation.

Specifically, we first used the reduce program in Amber 16 to add hydrogens to gefitinib and erlotinib. Then the antechamber program was applied to assign atomic charges and atom types for them. After that, the LEaP tool in Amber was used to generate the coordinate and topology files for the EGFR mutant-TKI complex. The Amber force fields protein.ff14SB and gaff2 were loaded and the EGFR mutant was loaded and combined with gefitinib or erlotinib to generate a single UNIT. After neutralizing the UNIT by adding Cl- or Na + ions, a solvent environment was created with the TIP3P water model and a truncated octahedral water box was used with a 10-Å buffer around the solute in each direction. At this point, the saveamberparm command in the LEaP tool can be used to save the coordinate and topology files for further processing.

After this preparation the simulation program pmemd can start the MD simulations. First a 1000-step energy minimization on the system was utilized to remove possible bad contacts within the system. Then, the system was heated from 0 K to 300 K over 50 ps. A density equilibration for 50 ps and a constant-pressure equilibration for 500 ps followed. For minimization, heating and density equilibration, a weak restraint with a weight of 2 (in kcal/mol-Å^2) is applied on all atoms of the solute. The equilibration of the system was validated by observing the stability of the temperature, density, energy, and root mean square deviation (RMSD) of the system.

Production MD simulations of 2 ns were performed at constant temperature and pressure. A trajectory of 1000 frames was obtained for each EGFR mutant-gefitinib/erlotinib complex.

### Interaction fingerprint calculation

**Fig. 6:**
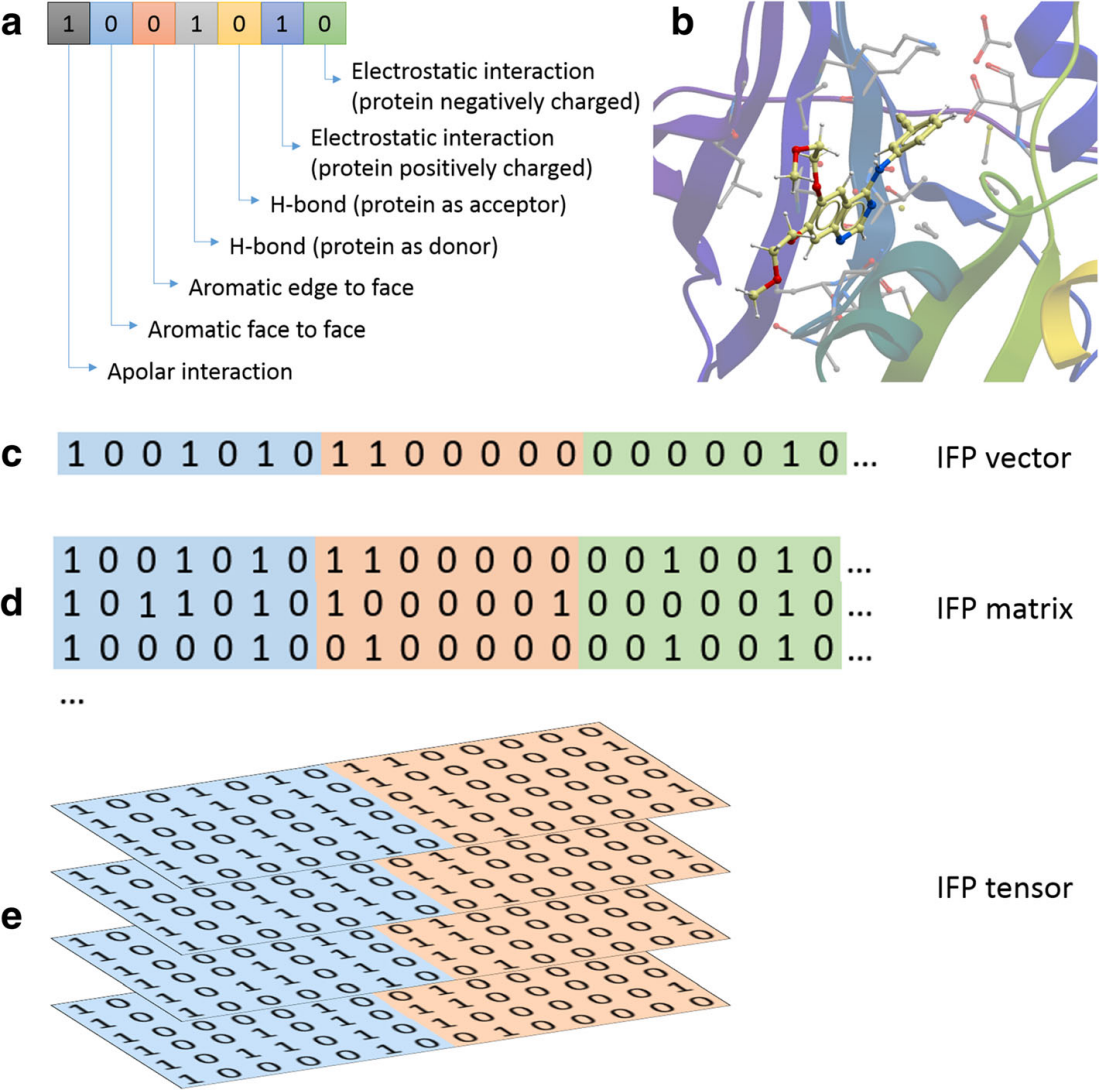
Interaction fingerprint. **a** Seven bits that represent seven different interactions for each residue. In the diagram, 1 means the interaction exists while 0 means the interaction does not exist. **b** Example of WT EGFR-erlotinib interactions (PDB: 1M17). The 3D figure was generated using Molsoft MolBrowser 3.8–5 (http://www.molsoft.com/). **c** For each frame in the MD trajectory, we can combine the 7-bit IFP of all residues to obtain its IFP vector. **d** For all frames in the MD trajectory of each complex we can produce an IFP matrix. This IFP matrix can be considered as the binding mode of this EGFR mutant with the specific TKI. **e** Combining these IFP matrices of multiple EGFR mutant-TKI complexes, we can obtain a third order IFP tensor

Our calculation of the interaction fingerprint (IFP) for each EGFR mutant-TKI complex is based on the PyPlif software [60], which is a python implementation of IFP. Seven different types of interactions for each residue are encoded (Fig. 6(a)), including Apolar (van der Waals), aromatic face to face, aromatic edge to face, hydrogen bond (protein as hydrogen bond donor), hydrogen bond (protein as hydrogen bond acceptor), electrostatic interaction (protein positively charged) and electrostatic interaction (protein negatively charged).

For each frame in the MD trajectory, we can combine the 7-bit IFP of all residues to obtain its IFP vector (Fig. 6(c)). For all frames in the MD trajectory of each complex, we can produce an IFP matrix (Fig. 6(d)). This IFP matrix can be considered as the binding mode of this EGFR mutant with the specific TKI. Combining these IFP matrices of multiple EGFR mutant-TKI complexes, we can obtain a third order IFP tensor (Fig. 6(e)).

### MPCA

MPCA [48] is a multilinear equivalent of PCA. Given a set of training tensor samples $\left\{{\mathcal{X}}_m\in {\mathbb{R}}^{I_1\times {I}_2\times \dots \times {I}_N},m=1,2,\dots, M\right\}$, where *I*_*n*_ is the n-mode dimension of the tensor, MPCA determines a multilinear transformation $\left\{{U}^{(n)}\in {\mathbb{R}}^{I_n\times {P}_n},n=1,2,\dots, N\right\}$ that maps the original tensor space ${\mathbb{R}}^{I_1}\bigotimes {\mathbb{R}}^{I_2}\dots \otimes {\mathbb{R}}^{I_N}$ into a tensor subspace ${\mathbb{R}}^{P_1}\bigotimes {\mathbb{R}}^{P_2}\dots \otimes {\mathbb{R}}^{P_N}$ (with *P*_*n*_ < *I*_*n*_, for *n* = 1, 2, …, N):

1 $$ {\mathcal{Y}}_m={\mathcal{X}}_m{\times}_1{U}^{(1)^T}{\times}_2{U}^{(2)^T}\dots {\times}_N{U}^{(N)^T},\mathrm{m}=1,2,\dots, \mathrm{M} $$

In other words, the MPCA objective is to determine the *N* projection matrices that maximize the total tensor scatter, so that the projected tensor objects $\left\{{\mathcal{Y}}_m\in {\mathbb{R}}^{P_1\times {P}_2\times \dots \times {P}_N},m=1,2,\dots, M\right\}$ preserve most of the variation observed in the original data:

2 $$ \left\{{U}^{(n)},n=1,2,\dots, N\right\}=\arg \max \sum \limits_{m=1}^M{\left\Vert {\mathcal{Y}}_m-\overline{\mathcal{Y}}\right\Vert}_F^2 $$

where $\sum \limits_{m=1}^M{\left\Vert {\mathcal{Y}}_i-\overline{\mathcal{Y}}\right\Vert}_F^2$ is a measure of the variation, or the total tensor scatter of all tensor samples. $\overline{\mathcal{Y}}$ is the mean tensor given by $\overline{\mathcal{Y}}=\left(\frac{1}{M}\right)\sum \limits_{m=1}^M{\mathcal{Y}}_m$.

### MPCA-based tensor object recognition

MPCA-based tensor object classification was employed to verify that the extracted IFP features were robust for the prediction of drug sensitivity. The recognition system used here was based on [48] and there were three main modules, preprocessing, feature extraction and classification.

#### A. Preprocessing

**Fig. 7:**
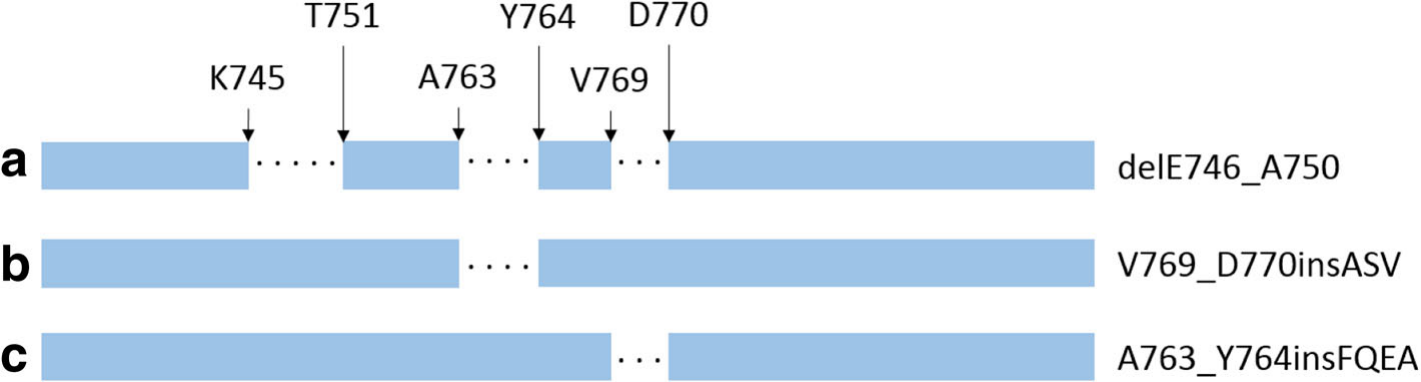
Example of normalizing the IFPs of three EGFR mutants to the same length by adding zeros. **a** For delE746_A750, 35 (5×7, where 7 means the 7 bits fingerprint for each residue) zeros are added between residues K745 and T751, due to the deletions of delE746_A750, 28 (4×7) zeros are added between residues A763 and Y764, due to the insertions of A763_Y764insFQEA, and 21 (3×7) zeros are added between residues V769 and D770, due to the insertions of V769_D770insASV. **b** For V769_D770insASV, 28 (4×7) zeros are added between residues A763 and Y764, due to the insertions of A763_Y764insFQEA. **c** For A763_Y764insFQEA, 21 (3×7) zeros are added between residues V769 and D770, due to the insertions of V769_D770insASV

MPCA only accepts tensor samples of the same dimensions. However, the 30 EGFR mutants have various number of residues and their corresponding IFPs have different lengths. We need to normalize all IFPs to the same length, which was done by adding zeros to proper positions of the IFPs of all EGFR mutants. As an example, we consider three EGFR mutants delE746_A750, V769_D770insASV and A763_Y764insFQEA (Fig. 7). For delE746_A750, 35 (5×7, where 7 means the 7 bits fingerprint for each residue) zeros are added between residues K745 and T751, due to the deletions of delE746_A750, 28 (4×7) zeros are added between residues A763 and Y764, due to the insertions of A763_Y764insFQEA, and 21 (3×7) zeros are added between residues V769 and D770, due to the insertions of V769_D770insASV. For V769_D770insASV, 28 (4×7) zeros are added between residues A763 and Y764, due to the insertions of A763_Y764insFQEA. For A763_Y764insFQEA, 21 (3×7) zeros are added between residues V769 and D770, due to the insertions of V769_D770insASV. Then, the IFPs of these three EGFR mutants will have the same length. The length-normalized tensor samples are then centered by subtracting the mean tensor of all tensor samples.

#### B. Feature extraction

MPCA is an unsupervised technique and the variation captured in the projected tensor subspace includes both within-class and between-class variation. For classification, a feature selection strategy [48], which enlarges the between-class variation and lessens the within-class variation, should be applied. Specifically, the class discriminability Γ is first calculated based on Eq. (3).

3 $$ {\Gamma}_{p1,p2,\dots, pN}=\frac{\sum \limits_{c=1}^C{N}_c\bullet {\left[{\overline{\mathcal{Y}}}_c\left(p1,p2,\dots, pN\right)-\overline{\mathcal{Y}}\Big(p1,p2,\dots, pN\Big)\right]}^2}{\sum \limits_{m=1}^M{\left[{\mathcal{Y}}_m\left(p1,p2,\dots, pN\right)-{\overline{\mathcal{Y}}}_{c_m}\Big(p1,p2,\dots, pN\Big)\right]}^2} $$

where ${\mathcal{Y}}_m$ is the projected tensor of ${\mathcal{X}}_m$, $\overline{\mathcal{Y}}$ and ${\overline{\mathcal{Y}}}_c$ are the mean tensors of all tensor samples and tensor samples in class c, respectively. C is the number of classes, M is the total number of samples, *N*_*c*_ is the number of samples for class c, and *c*_*m*_ is the class label for the tensor sample ${\mathcal{X}}_m$.

Then, the projected tensor ${\mathcal{Y}}_m$ is rearranged into a feature vector *y*_*m*_, in descending order according to the class discriminability Γ, and the first H most discriminative components of *y*_*m*_ are kept.

#### C. Classification

To verify that our extracted features are robust for the prediction of the sensitivity of each EGFR mutant to the drugs gefitinib or erlotinib, we performed classification experiments (with 10-fold cross-validation) using the 5 most commonly used classifiers available in Weka 3.8.0 [61], NaiveBayes, Logistic (logistic regression), RandomForest, libSVM (Support Vector Machine) and IBK (KNN, k-Nearest Neighbor). For RandomForest, we set the number of iterations to be performed as 500. For IBK we set the number of neighbor to use as 5. All other parameters are set to default values.

## Additional files

Additional file 1: **Figure S1.** (A) The temperature, (B) density, (C) energy and (D) backbone RMSD of the delE746_A750-gefitinib complex as functions of time. The system finally reaches a stable state after a series of equilibration operations. **Figure S2.** Class discrimination power of projected tensor features. (A) Class discrimination power of all projected tensor features of EGFR mutant-gefitinib complexes. (B) Class discrimination power of the first 30 most discriminative projected tensor features of EGFR mutant-gefitinib complexes. (C) Class discrimination power of all projected tensor features of EGFR mutant-erlotinib complexes. (D) Class discrimination power of the first 30 most discriminative projected tensor features of EGFR mutant-erlotinib complexes. (DOCX 115 kb)

Additional file 2: The list of extracted 20 features for EGFR mutant-gefitinib and -erlotinib complexes. The first column is the mutation name. The last column is the response level to gefitinib or erlotinib. The first row is the index of features. (XLSX 27 kb)

## Abbreviations

ATP: Adenosine triphosphate
CM: Comparative modeling
COSMIC: Catalogue of somatic mutation in cancer
EGFR: Epidermal growth factor receptor
IFP: Interaction fingerprint
KNN: K-nearest neighbors
MD: Molecular dynamics
MPCA: Multilinear principal component analysis
NSCLC: Non-small cell lung cancer
PCA: Principal component analysis
RMSD: Root mean square deviation
SVM: Support vector machine
TKI: Tyrosine kinase inhibitor
TPP: Targeted projection pursuit
WT: Wild type
