# Supplementary material for: Prediction of sensitivity to gefitinib/erlotinib for EGFR mutations in NSCLC based on structural interaction fingerprints and multilinear principal component analysis
Source: BMC Bioinformatics. 2018 Mar 7;19:88. doi: 10.1186/s12859-018-2093-6 (PMC5842518; doi:10.1186/s12859-018-2093-6)
Supplement: Supplementary file 1 — Figure S1. (A) The temperature, (B) density, (C) energy and (D) backbone RMSD of the delE746_A750-gefitinib complex as functions of time. The system finally reaches a stable state after a series of equilibration operations. Figure S2. Class discrimination power of projected tensor features. (A) Class discrimination power of all projected tensor features of EGFR mutant-gefitinib complexes. (B) Class discrimination power of the first 30 most discriminative projected tensor features of EGFR mutant-gefitinib complexes. (C) Class discrimination power of all projected tensor features of EGFR mutant-erlotinib complexes. (D) Class discrimination power of the first 30 most discriminative projected tensor features of EGFR mutant-erlotinib complexes. (DOCX 115 kb) [file 12859_2018_2093_MOESM1_ESM.docx]

**
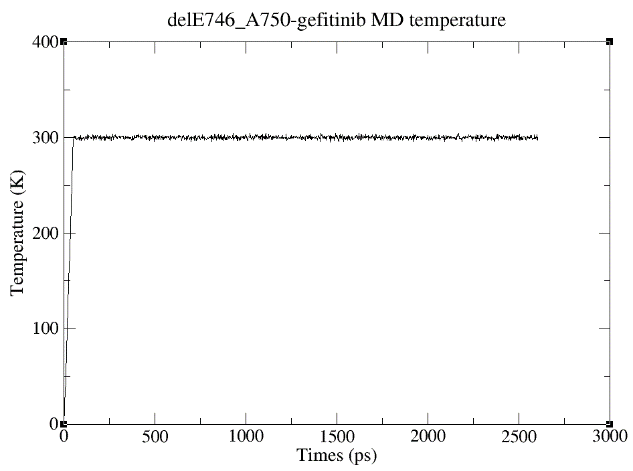

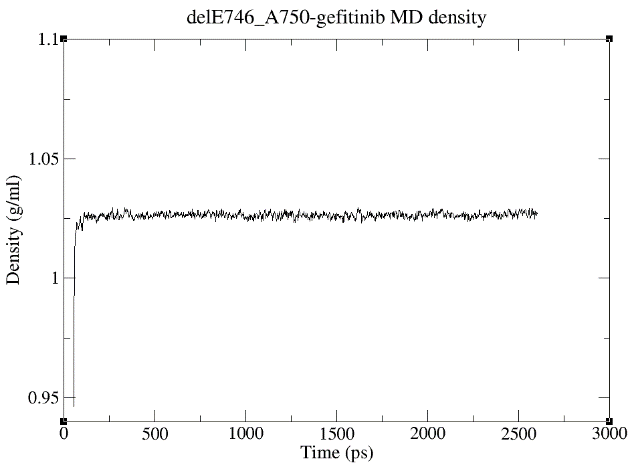
**

(A) (B)

**
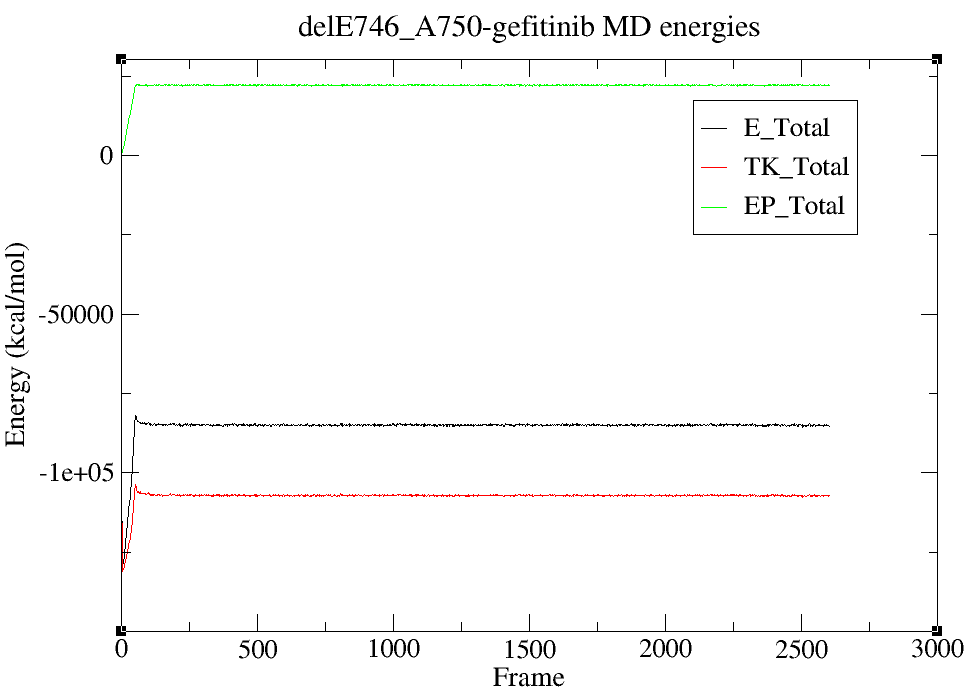

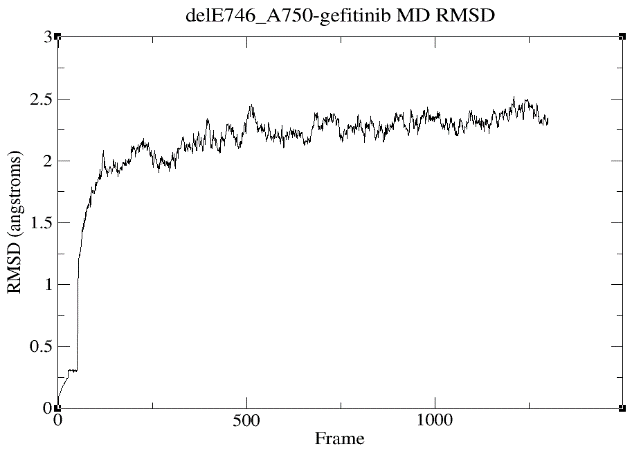
**

(C) (D)

**Supplementary Figure S1.** (A) The temperature, (B) density, (C) energy and (D) backbone RMSD of the delE746_A750-gefitinib complex as functions of time. The system finally reaches a stable state after a series of equilibration operations.


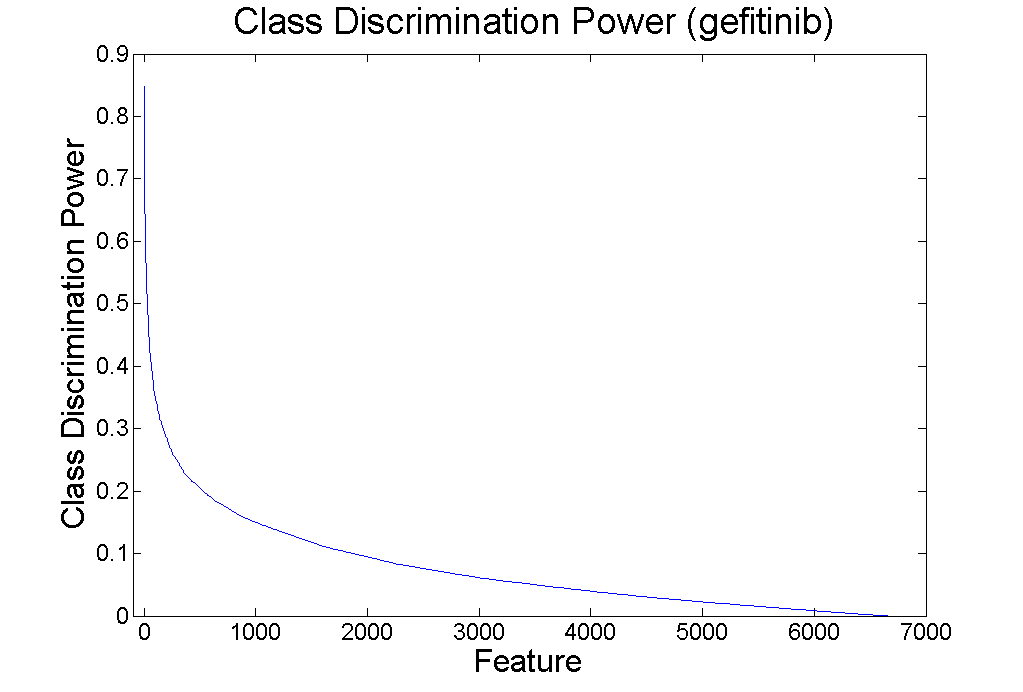

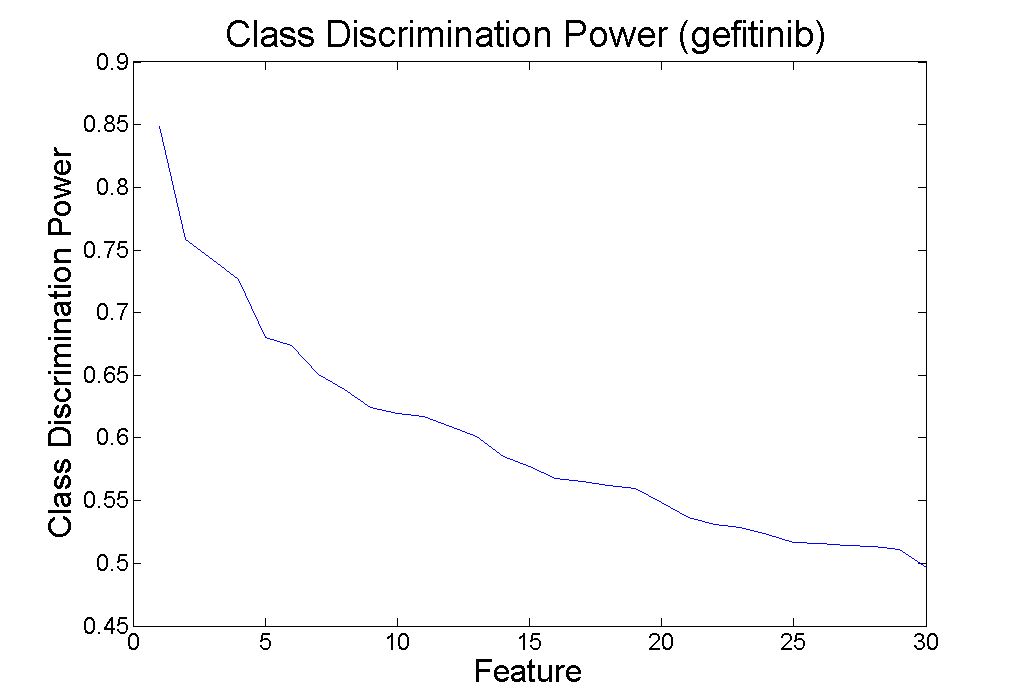


(A) (B)


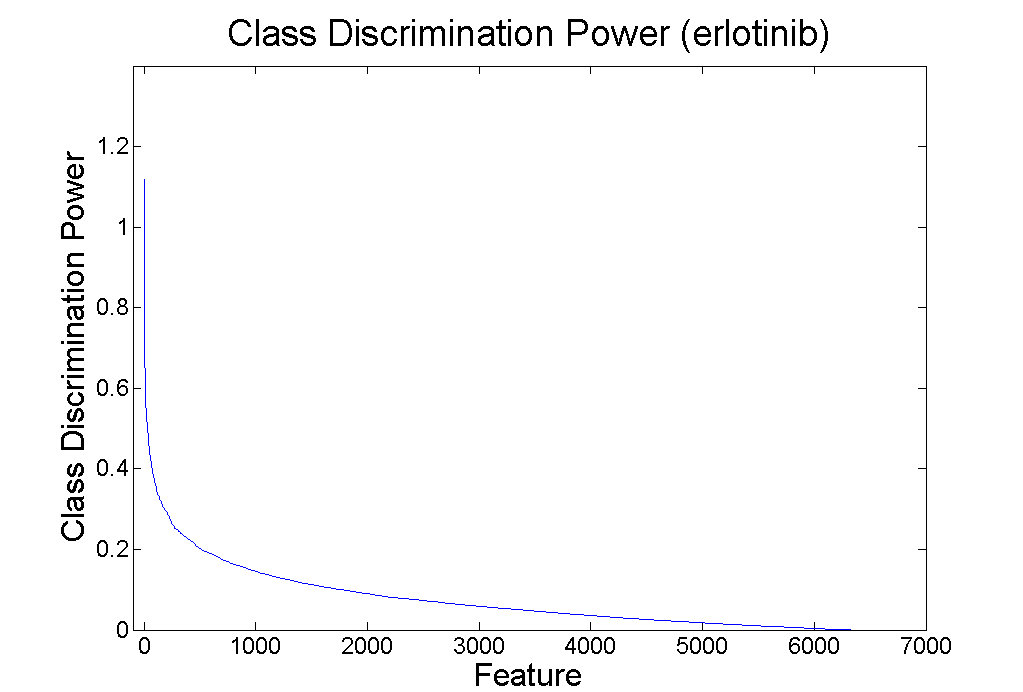

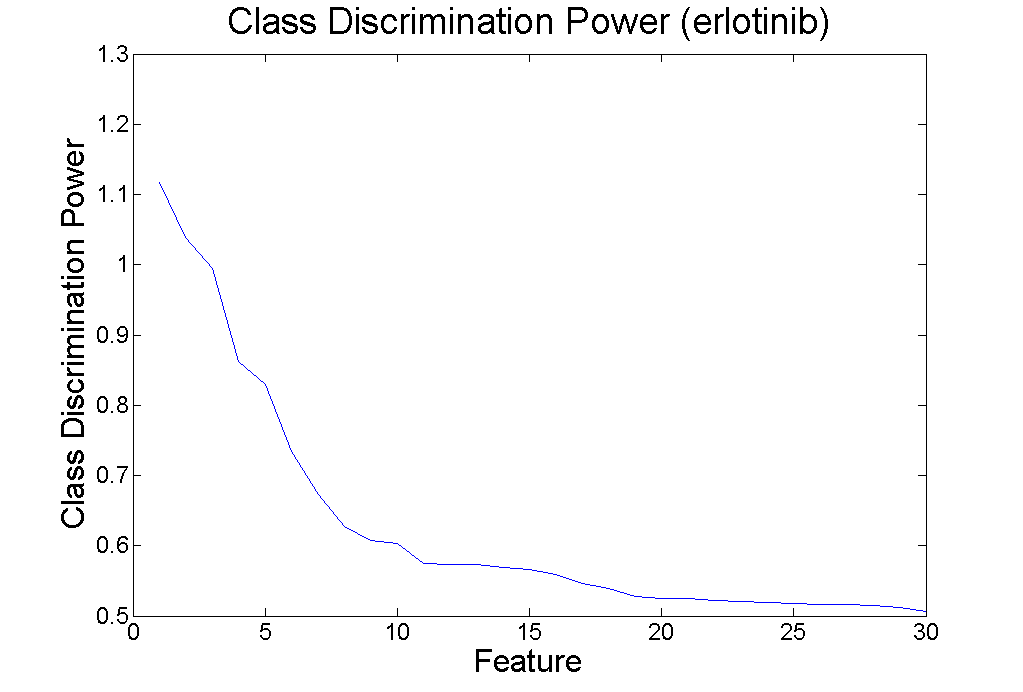


(C) (D)

**Supplementary Figure S2.** Class discrimination power of projected tensor features. (A) Class discrimination power of all projected tensor features of EGFR mutant-gefitinib complexes. (B) Class discrimination power of the first 30 most discriminative projected tensor features of EGFR mutant-gefitinib complexes. (C) Class discrimination power of all projected tensor features of EGFR mutant-erlotinib complexes. (D) Class discrimination power of the first 30 most discriminative projected tensor features of EGFR mutant-erlotinib complexes.
